# Maternal Early Life Adversity and Infant Stress Regulation: Intergenerational Associations and Mediation by Maternal Prenatal Mental Health

**DOI:** 10.1007/s10802-022-01006-z

**Published:** 2022-12-12

**Authors:** Margot E Barclay, Gabrielle R Rinne, Jennifer A Somers, Steve S Lee, Mary Coussons-Read, Christine Dunkel Schetter

**Affiliations:** 1 University of California, Los Angeles, USA; 2 University of Colorado, Colorado Springs, USA

**Keywords:** Early life adversity, Intergenerational transmission, Infant stress regulation, Maternal mental health, Pregnancy

## Abstract

Early life adversity is a potent risk factor for poor mental health outcomes across the lifespan, including offspring vulnerability to psychopathology. Developmentally, the prenatal period is a sensitive window in which maternal early life experiences may influence offspring outcomes and demarcates a time when expectant mothers and offspring are more susceptible to stressful and salutary influences. This prenatal plasticity constituted the focus of the current study where we tested the association of maternal early life adversity with infant stress regulation through maternal prenatal internalizing symptoms and moderation by prenatal social support. Mother-infant dyads (n = 162) were followed prospectively and mothers completed assessments of social support and depressive and anxiety symptoms across pregnancy. Infants completed standardized stress paradigms at one month and six months. There were several key findings. First, maternal prenatal depressive symptoms significantly mediated predictions of infant cortisol reactivity to the heel stick at one month from maternal early life adversity: specifically, maternal early life adversity positively predicted depressive symptoms in pregnancy, which in turn predicted dampened infant cortisol reactivity. Second, prenatal social support did not significantly moderate predictions of depressive or anxiety symptoms in pregnancy from maternal early life adversity nor did it alter the associations of maternal depressive or anxiety symptoms with infant stress regulation. These results suggest that maternal prenatal mental health is a key mechanism by which maternal early life adverse experiences affect offspring risk for psychopathology. We discuss potential clinical and health implications of dysregulated infant cortisol reactivity with respect to lifespan development.

**Supplementary Information:**

The online version contains supplementary material available at 10.1007/s10802-022-01006-z

Early life adversity increases risk for negative mental and physical health outcomes in the individual *and* their offspring (Bowers & Yehuda, 2015; Narayan et al., 2021; Roubinov et al., 2021). Encompassing a range of experiences spanning family dysfunction (e.g., exposure to violence, parental substance use), abuse (e.g., physical, emotional, sexual) and neglect (e.g., physical, emotional; Felitti et al., 1998), childhood adversity accounted for nearly 30% of the risk for adult psychiatric disorders based on nationally representative data from 21 countries (Kessler et al., 2010). Early life adversity has generational consequences as suggested by recent evidence that offspring of parents who experienced childhood adversity were more likely to exhibit socio-emotional and behavioral problems as well as psychopathology (Narayan et al., 2021). Despite these robust associations, early life adversity does not necessarily confer poor outcomes; that is, some individuals will demonstrate resilience to psychopathology (Masten & Cicchetti, 2016) supported through factors such as social support and coping abilities (Atzl et al., 2019). Thus, it is imperative to understand the pathways by which early childhood adversity influences subsequent generations as well as potential deviations from these pathways toward more adaptive, resilient outcomes.

Theoretically, *developmental cascades* refer to the cumulative consequences secondary to transactions between individuals and systems in development and heuristically positions the diverse processes through which key interactions are thought to affect development across generations (Masten & Cicchetti, 2016). Evidence in support of developmental cascades suggests that parental early life experiences of adversity increase risk of developmental delays in toddlers (Folger et al., 2018), disruptive behaviors in school-age children (Schickedanz et al., 2018), and psychopathology symptoms across the lifespan (Lyons-Ruth et al., 2006), likely through biological and behavioral pathways. For example, early traumatic events can be biologically embedded in individuals and their future offspring by altering stress-response systems. This chain reaction can shape brain development, stress regulation, and increase vulnerability to psychopathology onset (Cicchetti, 2002; Cicchetti & Cannon, 1999; Gunnar & Quevedo, 2007; Lupien et al., 2006); McEwen & Stellar, 1993; Meaney, 2010; Shonkoff, Boyce, & McEwen, 2009). Given that developmental cascades potentiate outcomes, prospectively, intergenerationally, and across multiple sensitive periods, resilience frameworks have described the *positive* consequences of naturally-occurring and intervention-induced cascades that may interrupt negative cascades and/or promote positive outcomes (Masten & Cicchetti, 2016). The prenatal period represents a potentially sensitive period whereby the expectant mother and offspring undergo substantial and rapid developmental changes that increase susceptibility not only to stress but also to salutary influences (Davis & Narayan, 2020). The current study focused centrally on this prenatal plasticity by examining risk and resilience cascades from maternal early life adversity to infant stress regulation.

Stress regulation reflects biologically-based tendencies to distress that emerge early in life and encompasses reactivity to and recovery from stress (Engel & Gunnar, 2020). There is increasing evidence that stress regulation originates in intergenerational processes (Spry et al., 2022). For example, maternal experiences prior to conception, including in her own childhood, predicted offspring emotional and physiological responses to stress (see Spry et al., 2022). Importantly, stress dysregulation in the form of heightened and dampened reactivity to stress may confer vulnerability to psychopathology across the lifespan (Davis et al., 2018; Koss & Gunnar, 2018; Thomas-Argyriou et al., 2021). There are several plausible pathways through which maternal early life experiences could influence offspring stress regulation, including social pathways (e.g., parenting), biological pathways (e.g., alterations to stress physiology), and shared environmental or genetic factors (Bowers & Yehuda, 2015). The prenatal period is a particularly important period in which preconception maternal experiences may influence offspring stress regulation through biological pathways, specifically prenatal programming of the hypothalamic-pituitary-adrenal (HPA) axis, a primary biological stress response system (Bowers & Yehuda, 2015; Roubinov et al., 2021; Davis & Narayan, 2020). The HPA axis undergoes a rapid and ordered sequence of development in the prenatal period, with differentiation of the hypothalamus occurring as early as 10 weeks gestation and development of the adrenal cortex continuing through 30 weeks gestation. Because the HPA axis develops rapidly over the course of gestation, it is particularly sensitive to environmental inputs such that variations in fetal exposures to stress hormones and maternal stress signals across gestation may affect offspring stress regulation over the lifespan (Howland et al., 2017).

## Maternal Early Life Adversity and Offspring Stress Regulation

Biological changes secondary to maternal experiences of childhood adversity may shape the development of the fetal HPA axis. Adversity-linked epigenetic changes and changes in maternal biological stress responses expose offspring to more maternal stress hormones in utero and thus potentiate neuroendocrine changes in childhood in both stress and non-stress conditions (Bowers & Yehuda, 2015; Davis & Narayan, 2020; Yehuda & Lerner, 2018). For example, maternal early life adversity predicted infant dysregulated emotional reactivity and recovery from the Still Face paradigm (Hipwell et al., 2019); similarly, greater maternal early life adversity positively predicted greater infant cortisol reactivity to a stressor task at six months indirectly through maternal cortisol awakening response in pregnancy (Thomas et al., 2018). However, other studies observed that maternal early life adversity or trauma were unrelated to infant cortisol reactivity (Bosquet Enlow et al., 2011; Brand et al., 2010). Thus, the precise associations between maternal early life adversity and infant stress regulation are unclear, necessitating designs that afford strong inferences about potential mediating processes.

## Mediating Pathways: Maternal Internalizing Symptoms During Pregnancy

Depressive and anxiety symptoms in pregnancy are among the most common pregnancy complications (Dunkel Schetter, 2012; Gavin et al., 2005), and individuals with a history of family stress and dysfunction may be particularly susceptible to depressive and anxiety symptoms in pregnancy (for a review, see Racine et al., 2021). In turn, internalizing symptoms in pregnancy are known to predict offspring HPA axis dysregulation, emotional problems, and behavioral problems through adolescence even when controlling for postnatal maternal mental health (e.g., Davis et al., 2018; Rogers et al., 2020; Tirumalaraju et al., 2020). Within the context of developmental cascades, maternal mental health problems in pregnancy may therefore be a key risk pathway linking maternal early life adversity to offspring stress regulation, likely operating through changes to the intrauterine milieu and fetal exposures that have previously been linked to maternal depressive and anxiety symptoms in pregnancy (e.g., Peterson et al., 2020; Ramos et al., 2022; Rinne et al., 2022). Consistent with this formulation, another study reported that maternal early life adversity predicted poor maternal mental health and attachment insecurity in pregnancy which in turn predicted higher offspring internalizing and externalizing problems at age five among nearly 2,000 mother-child pairs (Cooke et al., 2019). Despite evidence that maternal depressive and anxiety symptoms in pregnancy predicted dysregulated cortisol reactivity to acute stress (see Howland et al., 2017 for a review), studies have not yet tested maternal internalizing symptoms in pregnancy as a pathway between maternal early life adversity and offspring HPA axis regulation. Studies that specifically test whether maternal depressive and anxiety symptoms in pregnancy explain the association between maternal early life adversity and infant cortisol regulation are necessary to elucidate pathways implicated in the intergenerational transmission of stress regulation and to inform targets of intervention.

## Protective Factor: Social Support During Pregnancy

As noted previously, hypothesized risk-outcome associations in maternal and offspring development include discontinuities that may facilitate delivery of targeted interventions (Masten & Cicchetti, 2016). The social context may clarify the intergenerational association of maternal early life adversity with offspring mental and physical health problems, including its potential mediation by prenatal mental health (Racine et al., 2020). Pregnancy is a sensitive period as mothers and infants are highly receptive to positive environmental influences (Davis & Narayan, 2020), including resilience-promoting factors (Atzl et al., 2019) such as social support (Liu et al., 2022; Collins et al., 1993; Hetherington et al., 2018). Therefore, as a second aim, we tested prenatal social support as a protective factor against internalizing symptoms for mothers and dysregulated stress responses for infants.

Social support in pregnancy may benefit mothers and infants through multiple pathways. From a theoretical perspective, social support may serve as a resilience-promoting factor by modifying the associations between early adversity and subsequent mental health outcomes or stress physiology, particularly HPA axis regulation (e.g., social buffering of the HPA axis, social baseline theory; Beckes & Coan 2012; Gunnar, 2017; Hostinar & Gunnar, 2013). Consistent with such theories, several recent investigations provide support for a central role of social support in pregnancy as a protective factor in the context of intergenerational transmission. First, perceived partner support during pregnancy predicted favorable infant cortisol responses to stress through improved maternal depression in pregnancy and enhanced mother-infant interactions postpartum (Thomas et al., 2017). Second, the association of infant temperament with infant cortisol reactivity was attenuated among mothers who received high emotional or tangible social support from their social network in pregnancy, suggesting that prenatal social support improved outcomes over and above maternal mental health (Luecken et al., 2015). Lastly, supportive prenatal environments may mitigate intrauterine alterations secondary to early adversity and prenatal psychological distress: social support was inversely associated with maternal and placental-fetal stress hormone levels in pregnancy (Giesbrecht et al., 2013; Hahn-Holbrook et al., 2013). Thus, social support in pregnancy may buffer effects of maternal early life experiences and distress on infant outcomes, although this has been infrequently explored with respect to infant stress regulation as an outcome. Of note, these protective effects are not limited to a certain type of social support and have been observed across different types and sources of social support.

## The Current Study

**Fig. 1:**
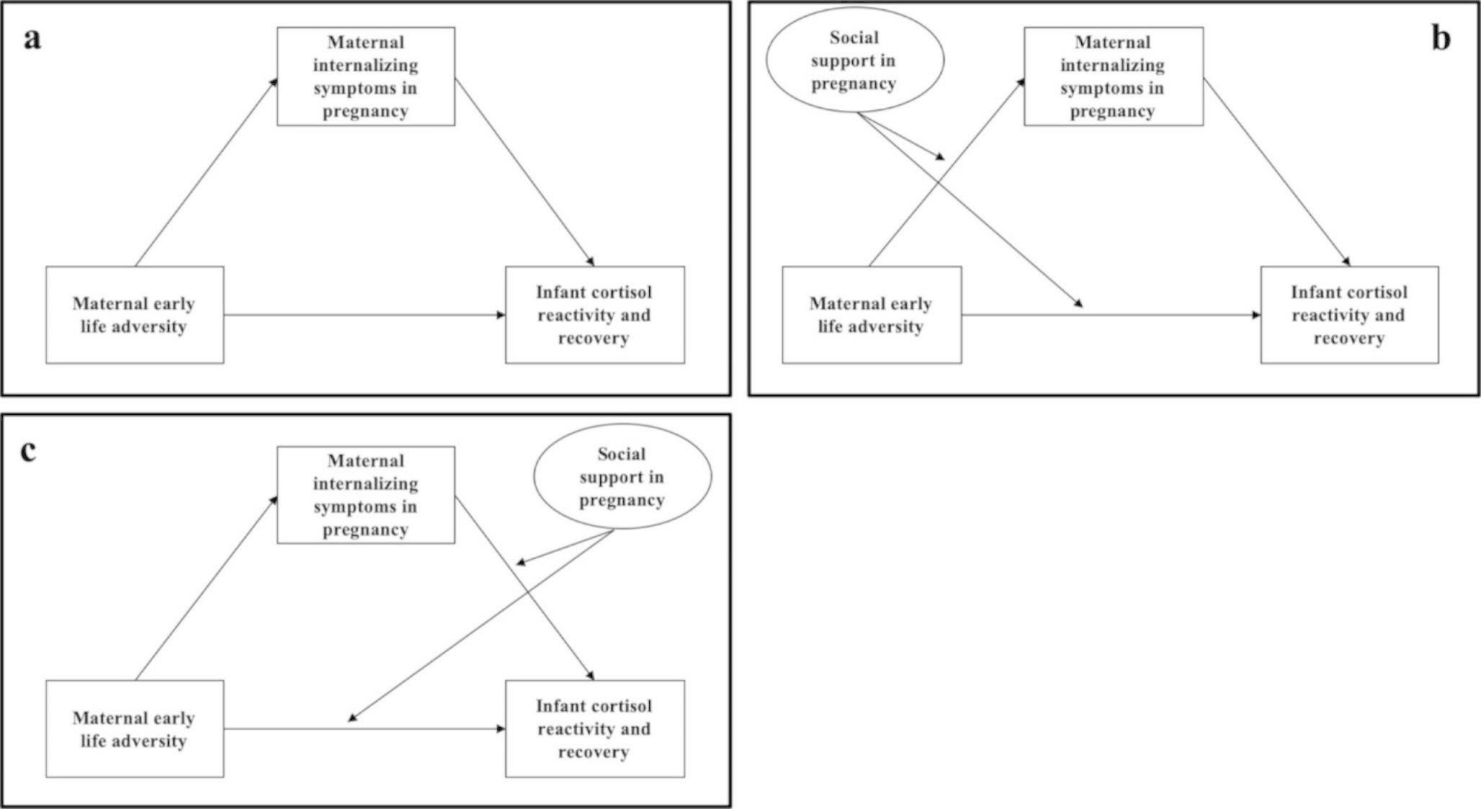
Primary research hypotheses were evaluated using structural equation models (SEMs) represented above using standard SEM notation; observed variables are shown as rectangles and latent variables are shown as ovals. The first SEM evaluated the base model (Panel A) whereby maternal early life adversity influences infant cortisol responses at one month and six months via maternal prenatal internalizing symptoms. Two alternative models to evaluate the moderating role of maternal prenatal social support tested: (1) maternal social support as a buffer against the effect of maternal early life adversity on infant cortisol regulation via reduced maternal prenatal internalizing symptoms (mediated moderation; Panel B) and (2) maternal social support as a buffer against the effect of maternal prenatal internalizing symptoms on infant cortisol stress regulation (moderated mediation; Panel C). Social support was derived from a latent variable.

To identify early risk and resilience-promoting factors during the prenatal period that will guide intervention and prevention efforts, the aims of this study were two-fold: (1) to test the intergenerational association of maternal early life adversity with offspring stress reactivity and recovery derived from infant cortisol responses to stress paradigms at one month and six months of age, including mediation by maternal depressive and anxiety symptoms in pregnancy (Fig. 1, Panel A) and (2) to test moderation by maternal prenatal social support. In our first aim, we hypothesized that greater maternal early life adversity would predict more anxiety and depressive symptoms in pregnancy which in turn would predict dysregulated infant cortisol reactivity and recovery.

We considered two alternative models for the second aim: the first tested if social support modified the association of maternal early life adversity with infant cortisol reactivity and recovery via reduced maternal anxiety and depressive symptoms (i.e., mediated moderation; Fig. 1, Panel B). The second model tested prenatal social support as a buffer against predictions of infant cortisol reactivity and recovery from maternal anxiety and depressive symptoms in pregnancy (i.e., moderated mediation; Fig. 1, Panel C). We tested both pathways based on evidence that social support in pregnancy may benefit mothers and infants through multiple pathways. For instance, social support during pregnancy has been found to indirectly predict favorable infant cortisol responses to stress through improved maternal mental health in pregnancy (mediated moderation; Thomas et al., 2017). Conversely, social support may modify associations between maternal mental health and infant outcomes potentially through buffering against the downstream effects of maternal mental health on alterations to prenatal stress physiology (moderated mediation; Leucken et al., 2015; Thomas et al., 2018). We hypothesized that social support would serve as a protective factor in this intergenerational association and constitute a potentially modifiable intervention target for mothers and infants. Due to the limited research in this area and plausible theoretical and empirical justification for both pathways, we did not propose specific hypotheses regarding the manner in which social support would modify each mediational pathway.

## Method

### Participants and Procedure

233 pregnant women were enrolled in Healthy Babies Before Birth (HB3), a longitudinal study designed to test the impact of antenatal maternal mood on birth outcomes and infant development. Participants were 18 years of age or older, with singleton intrauterine pregnancies, who were receiving care at prenatal clinics and private practices in Denver, Colorado and Los Angeles, California. Participants were recruited prior to their 12th week of gestation at prenatal appointments. Trained study team staff identified pregnant women at prenatal care appointments, and if women were eligible, they were invited to participate in the study. Most of the recruitment was through direct patient contact in prenatal clinics at the major medical centers, but some also came from placing study brochures in prenatal care settings. In Los Angeles, participants were recruited at a major medical center where the affiliated prenatal clinics serve a range of women in terms of income. In Denver, participants were recruited at one prenatal clinic affiliated with a major medical center serving mostly low-income women. Across both sites, it is estimated that 22.9% of those who were eligible for the study and were approached enrolled in the study. Reasons to decline were time required, transportation, and lack of interest. Denver participants were included if they spoke English or Spanish as a primary language whereas participants in Los Angeles were English-speaking only. Exclusion criteria were current substance abuse diagnosis, HIV-positive status, current smoking, and multiple gestation. Study staff obtained written informed consent from all participants who expressed interest. Participants were compensated for each study visit.

The study consisted of three prenatal and three postnatal visits conducted with trained research staff. Participants were evaluated in early pregnancy (8–16 weeks gestation), mid-pregnancy (20–26 weeks gestation), late pregnancy (30–36 weeks gestation), 4–8 weeks postpartum, 5–7 months postpartum, and 11–13 months postpartum. Women reported on household income, household size, and number of previous live births at enrollment and reported on infant age and breastfeeding status at the postnatal visits. Per capita household income was calculated as household income divided by household size and adjusted for cost of living at each site. Infant birth outcomes (gestational age, birth weight, and Apgar scores at 5 minutes) were abstracted from medical records. Each institution’s Institutional Review Board approved all protocols and procedures at each study site (Cedars-Sinai Medical Center; University of Colorado).

The current study included data from the study visits in early pregnancy, mid pregnancy, late pregnancy, one month postpartum, and six months postpartum and only from those participants who completed the prenatal study visits and the visit one month postpartum (*n* = 162).^1^ Mean maternal age at enrollment was 32 years (*SD* = 6.13). Most participants self-identified as White (79.6%) and one-third identified as Hispanic/Latina (34.0%). Mean per capita annual household income adjusted for cost of living at each site was $32,772 (*SD* = $30,077). More than half of the sample was pregnant with their first child (59.3%). Most women were either married (73.5%) or in a relationship (22.2%) at enrollment. Half of the infants were male (50%). Mean infant age was 1.26 months (*SD* = 1.13 months) at the first postnatal visit and 5.62 months (*SD* = 1.01 months) at the second postnatal visit. Participant characteristics and demographics are presented in Table 1.

**Table 1:** Sample Demographics (n = 162)

	% (n)	Mean (*SD*), Range
Maternal age (years)	–	31.55 (6.13), 18–45
Ethnicity		
*Not Hispanic/Latina*	65.4 (106)	–
*Hispanic/Latina*	34.0 (55)	–
Race		
*White*	79.6 (129)	–
*Asian*	8.6 (14)	–
*Black/African American*	8.0 (13)	–
*Multiracial or other*	3.1 (5)	–
Per capita income (adjusted for cost of living)	–	$32,772 ($30,007), $0-$140,845
Parity	–	0.85 (1.12), 0–5
*Nulliparous*	59.3 (96)	–
*Primiparous*	40.7 (66)	–
Relationship status		
*Married*	73.5 (119)	–
*In a relationship*	22.2 (38)	–
*Single*	3.1 (5)	–
Education (years)	–	16.31 (3.34), 8–26
*Less than high school*	2.5 (4)	
*Completed high school*	12.3 (20)	
*Some college*	16.7 (27)	
*College degree or higher*	67.9 (110)	
Language preference		
*English*	95.7 (155)	–
*Spanish*	3.1 (5)	–
Infant sex		
*Female*	46.9 (76)	–
*Male*	50.0 (81)	–
Gestational age, at birth (weeks)	5.6 (9)	39.04 (1.61), 22–42
Birth weight (grams)	5.2 (8)	3,324 (617), 1,086 − 4,624
Apgar score (1 min)	–	7.78 (1.78), 1–9

*Note.* Participants selected one (or more) categories for race and one category for ethnicity. Some percentages do not add to 100 due to missing data.

^a^ % preterm (< 37 weeks gestation)

^b^ % low birthweight (< 2,500 g)

### Measures

#### Maternal Early life Adversity

In early pregnancy, participants reported the frequency of early life adversity with the Risky Families Questionnaire, which is a 10-item well-validated self-report measure of family climate and dysfunction including household chaos, household disorganization, emotional abuse, and physical abuse (Repetti et al., 2002; Taylor et al., 2004). Participants reported on the frequency of occurrence of each experience in childhood between the ages of 5 and 15 on a scale of 1 (*not at all*) to 5 (*very often*). Sample items included: “How often did a parent or other adult in the household swear at you, insult you, put you down, or act in a way that made you feel threatened?”; “How often would you say you were neglected while you were growing up, that is, left on your own to fend for yourself?”. Three items pertaining to feeling loved, physical affection, and household organization were reverse coded. All items were then summed with a range of scores from 10 to 50. Original psychometric validation of the Risky Families Questionnaire included validation with respect to interview-based measures of childhood adversity, and links between the Risky Families measure and health outcomes persisted when controlling for potential confounders, including neuroticism (Taylor et al., 2004). Cronbach’s alpha indicated excellent reliability in the current sample (alpha = 0.91).

#### Maternal Internalizing Symptoms

**Depressive symptoms.** At each visit, participants completed the Patient Health Questionnaire (PHQ-9; Kroenke et al., 2001), a screening, diagnosing, and monitoring measure of depression. Participants rated symptom frequency (past two weeks) on a scale of 0 (*not at all*) to 4 (*nearly every day*). Sample items included: “Over the last 2 weeks, how often have you had little interest or pleasure in doing things?”; “Over the last 2 weeks, how often have you had trouble concentrating on things, such as reading the newspaper or watching television?”. Items were summed and total scores ranged from 0 to 36. The validity of the PHQ-9 has been previously established in primary care and obstetric clinics. Cronbach’s alphas were acceptable at each timepoint in the current sample (alphas = 0.66–0.83).

**Anxiety symptoms.** At each study visit, participants completed the Overall Anxiety Severity and Impairment Scale (OASIS; Norman et al., 2006), a 5-item rating scale of anxiety frequency, intensity, behavioral avoidance, and functional impairment in the past week. Sample items included: “In the past week, how often have you felt anxious?”; “In the past week, how often did you avoid situations, places, objects, or activities because of anxiety or fear?”. Items were rated on a scale of 0 (*low*) to 4 (*high*) and then summed with total scores ranging from 0 to 20. The measure had good reliability at each timepoint in this sample (alphas = 0.80–0.87).

### Social Support

Participants self-reported social support in early pregnancy, mid pregnancy, and late pregnancy. In early pregnancy, participants rated seven items selected from the National Survey of American Life (Jackson et al., 2004) on the frequency of family support, frequency of non-spousal family support, frequency of friend support; perceived closeness to family and friends; and frequency of contact with family and friends (e.g., “How often do you help out people in your family -- including children, grandparents, aunts, uncles, in-laws and so on? Would you say very often, fairly often, not too often, or never?”). The Cronbach’s alpha was 0.80 for the family-related items and 0.72 for the friend-related items.

In mid pregnancy, participants reported on social support with the ENRICHD Perceived Social Support Instrument. The Perceived Social Support Instrument is a 7-item measure that assesses four defining attributes of social support: emotional, instrumental, informational, and appraisal (e.g., “Is there someone available to you whom you can count on to listen to you when you need to talk?”). Participants reported on the availability of social support from any source on a scale of 1 (*none of the time*) to 5 (*all of the time*). Items were summed for a total score of social support that ranged from 7 to 35. The Cronbach’s alpha was 0.83.

In late pregnancy, mothers rated six items of the frequency of overall support received from the baby’s father or their partner over the course of pregnancy (Collins et al., 1993; e.g., “Since you became pregnant, how often has the baby’s father [OR husband OR partner] listened to you and understood your problems or concerns?”). Scores ranged from 1 (*never*) to 5 (*always*). Items were summed for a total score of partner support, which ranged from 6 to 30. The Cronbach’s alpha in this sample was 0.86.

### Infant Salivary Cortisol

Infant salivary cortisol was collected during two standardized, developmentally appropriate stress paradigms (Gunnar et al., 2009). The heel stick paradigm was administered at one month and the Still Face paradigm was administered at six months to elicit cortisol responses to physical pain and social stress, respectively (see Gunnar et al., 2009 and Weinberg & Tronick, 1994 for additional task details). At one month, infant saliva samples were collected upon arrival to the lab prior to a heel stick blood draw and then 20 and 40 minutes after the heel stick blood draw. At six months, infant saliva samples were collected upon arrival to the lab prior to the Still Face paradigm and then 15 minutes, 30 minutes, and 45 minutes after the start of the Still Face paradigm. At both study visits, the stressor paradigms were the first tasks completed by the infant.

Following collection, saliva samples were frozen. Frozen samples were centrifuged for 15 minutes at 3000 rpm to extract samples and aliquoted into cryogenic storage vials (300-500ml aliquots) and frozen at -80 C until analysis. Cortisol concentrations (µg/dl) were determined using a commercial high sensitivity EIA kit (Salimetrics) according to the directions provided by the manufacturer. Samples were run in duplicate, and optical density at 450 nm was assessed using an automatic microplate reader (BioTek). The amount of cortisol in each sample was determined using the standard curve generated with each assay. Samples were run in large cohorts utilizing the same manufacturer’s lot to reduce assay drift and interassay variability. The mean of the duplicates was used as the unit of analysis for statistical evaluation of these data. The intra-assay CVs ranged from 7.13 to 10.72%.

Infant cortisol reactivity and recovery were captured with two delta (or difference) scores, consistent with prior literature (e.g., Irwin et al., 2021; Noroña-Zhou et al., 2020). Cortisol reactivity was calculated by subtracting the peak cortisol levels (20 to 30 minutes following stressor onset; Gunnar & White 2001) from baseline cortisol levels. Cortisol reactivity to the heel stick was calculated as the difference in cortisol levels at 20 minutes from baseline levels and cortisol reactivity to the Still Face was calculated as the difference in cortisol levels at 30 minutes from baseline levels. Cortisol recovery was calculated as the difference between cortisol levels 40 minutes after stressor onset and peak cortisol levels for the heel stick and the difference between cortisol levels 45 minutes after stressor onset and peak cortisol levels for the Still Face. Infant cortisol measures were log-transformed to account for non-normality (skewness > 2; kurtosis > 7; West et al., 1995) prior to calculating delta scores.

### Data Analytic Plan

#### Preliminary Analyses

**Latent constructs of maternal internalizing symptoms and social support in pregnancy.** We conducted two factor analyses, one of the anxiety and depressive variables and one of the nine social support variables. We conducted a factor analysis on the nine measures of social support used in the study to formulate a parsimonious index of social support over the course of pregnancy and to avoid increasing Type I error rate from conducting multiple analyses with one measure at one timepoint. Factor analysis also offers analytic advantages by identifying a latent construct that reflects underlying common variance shared by multiple measures (e.g., Watkins et al., 2018).

One-, two-, and three-factor solutions were evaluated with exploratory factor analysis with oblique geomin rotation to allow for correlated factors. Confirmatory factor analysis (CFA) evaluated the best-fitting models of prenatal maternal internalizing symptoms and social support. Given model complexity, saved factor scores were used in primary analyses.

### Potential Covariates

Covariates were selected based on empirical precedent, associations with primary study variables, and associations with missingness to satisfy missing at random assumptions of FIML (Collins et al., 2001). Maternal depressive and anxiety symptoms at the time of infant cortisol assessment were *a priori* covariates to isolate the effects of prenatal maternal internalizing symptoms specifically. Primiparity (one or more previous live births versus no previous live births), birthweight, gestational age at birth, Apgar score, infant sex, per capita household income, maternal race and ethnicity, maternal age, and study site were evaluated as covariates given links with primary study variables in prior research. Covariates were included in final models if associated with primary study variables or missingness on primary study variables.

Maternal ethnicity was associated with prenatal anxiety symptoms (*t*(76) = 1.99, *p* = .05) and missingness on prenatal anxiety symptoms (chi-squared(1) = 4.48, *p* = .03). Participants who identified as Hispanic/Latina reported lower prenatal anxiety symptoms (*M* difference = 2.82) and were more likely to have missing values for prenatal anxiety symptoms compared to those who did not identify as Hispanic/Latina. Higher per capita household income was associated with greater social support during pregnancy (*r* = .18, *p* = .02), infant cortisol reactivity at one month (*r* = -.20, *p* = .01), missingness on one month reactivity (*t*[154] = -2.68, *p* = .01) and recovery (*t*[137] = -3.12, *p* = .002), and missingness on six month reactivity (*t*[153] = -2.95, *p* = .003) and recovery (*t*[149] = -3.51, *p* < .001). Infants of older mothers were more likely to have missing cortisol reactivity and recovery data. Therefore, of the nine covariates evaluated for inclusion, we retained postnatal internalizing symptoms, maternal age, maternal ethnicity, and per capita income as covariates in primary models. Results of primary models did not change when covariates were included versus excluded; we present results of primary models adjusting for covariates.

### Primary Analyses

We employed structural equation modeling for primary analyses (see Fig. 1 for a conceptual overview). Each structural equation model separately analyzed infant outcomes at each timepoint and adjusted for maternal postnatal internalizing symptoms at the time of cortisol measurement as well as covariates of maternal age, ethnicity, and income.^2^

We employed M*plus* v.8.4 (Muthén & Muthén, 1998–2017 ) and analyzed all available values and full information maximum likelihood estimation (FIML) with robust standard errors. Missing data ranged from 0% on depressive and anxiety symptoms in pregnancy to 34% on infant cortisol reactivity at six months. FIML estimates are superior to pairwise or listwise deletion to manage missing data and are recommended when missing data exceeds 10% to reduce bias and maintain power. FIML is also recommended in the context of quantitative moderators (Enders, 2001; Enders & Bandalos, 2001). Maximum likelihood parameter estimates with standard errors that are robust to non-normality are computed using a sandwich estimator. All exogenous variables were grand mean-centered before forming product terms. Recommended cut-offs for fit indices included: RMSEA < 0.06, SRMR < 0.08, and CFI > 0.95 (West et al., 2012).

In the base model, mediation was tested by examining the indirect effect of maternal early life adversity on infant cortisol reactivity and recovery via maternal prenatal internalizing symptoms. Mediation of indirect effects was tested by examining the statistical significance of the indirect effect using 95% bootstrap confidence intervals (CIs). If the 95% CI does not contain 0, there is evidence of significant mediation. In the mediated moderation model, significant interactions (alpha < 0.05) were probed by testing the simple slopes of the effects of maternal early life adversity on prenatal internalizing symptoms at the mean and +/- 1 *SD* above and below the mean on maternal prenatal social support. In the moderated mediation model, we tested the conditional indirect effects of maternal early life adversity on infant cortisol reactivity and recovery via internalizing symptoms at the mean and +/-1 *SD* above and below the mean of maternal prenatal social support.

## Results

### Descriptive Statistics

Descriptive statistics and bivariate correlations for key study variables appear in Table 2. Higher maternal early life adversity significantly predicted more depressive symptoms in pregnancy (*r* = .31, *p* < .001) and lower social support in pregnancy (*r* = -.42, *p* < .001). Maternal depressive symptoms in pregnancy predicted higher infant cortisol reactivity (*r* = .29, *p* = .036) and lower infant recovery (*r* = -.31, *p* = .024) at six months. Greater maternal anxiety symptoms also significantly predicted higher infant cortisol reactivity (*r* = .37, *p* = .007) and lower recovery (*r* = -.38, *p* = .004) at six months.

**Table 2:** Means, standard deviations, and bivariate correlations

Variable	*M*	*SD*	1	2	3	4	5	6	7	8	9	10	11	12	13
1. Maternal early life adversity	20.48	9.05													
2. Prenatal depressive symptoms	12.63	7.28	0.31**												
3. Prenatal anxiety symptoms	10.53	7.52	0.14	0.47**											
4. Prenatal social support	0.00	0.93	− 0.42**	− 0.28**	− 0.24**										
5. Baseline infant cortisol 1 month	0.27	0.51	− 0.07	0.08	0.12	− 0.07									
6. +20 min. infant cortisol 1 month	0.35	0.30	− 0.04	− 0.01	0.08	− 0.04	0.69**								
7. +40 min infant cortisol 6 months	0.30	0.56	− 0.00	− 0.17	− 0.04	− 0.02	0.41**	0.66**							
8. Infant cortisol reactivity 1 month	0.52	0.83	− 0.04	− 0.11	0.02	0.21	− 0.34**	0.30*	0.06						
9. Infant cortisol recovery 1 month	-0.28	0.49	0.01	− 0.08	− 0.04	0.02	0.03	− 0.12	0.43**	− 0.37**					
10. Baseline infant cortisol 6 months	0.11	0.08	0.15	− 0.12	− 0.13	− 0.12	− 0.07	− 0.02	0.06	− 0.27	0.03				
11. +30 min infant cortisol 6 months	0.16	0.24	− 0.02	0.05	0.18	0.06	− 0.07	− 0.16	− 0.08	− 0.29	0.04	0.54**			
12. +45 min infant cortisol 6 months	0.12	0.12	− 0.04	0.01	0.08	− 0.03	− 0.05	0.04	0.04	− 0.23	0.00	0.35**	0.69**		
13. Infant cortisol reactivity 6 months	0.15	0.70	0.01	0.29*	0.37**	0.00	− 0.14	− 0.26	− 0.20	− 0.20	0.05	− 0.20	0.59**	0.50**	
14. Infant cortisol recovery 6 months	-0.20	0.53	− 0.17	− 0.31*	− 0.38**	− 0.04	0.13	0.33*	0.21	0.32	− 0.09	− 0.12	− 0.46**	0.05	− 0.54**

*Note.* Raw infant cortisol concentrations (µg/dl) are presented for ease of interpretation. Reactivity and recovery delta scores were calculated using log-transformed cortisol values.

* *p* < .05. ** *p* < .01.

Changes in mean infant cortisol levels are shown in Table 2. Mean infant cortisol levels significantly increased from baseline to 20 minutes after the heel stick at one month (reactivity; *t*(62) = 4.94, *p* < .001) and significantly decreased from 20 to 40 minutes after the heel stick (recovery; *t*(57) = -4.29, *p* < .001). Mean infant cortisol levels marginally increased from baseline to 30 minutes after the Still Face at six months (reactivity; *t*(57) = 1.66, *p* = .10). Mean cortisol levels significantly decreased from 30 to 45 minutes after the Still Face (recovery; *t*(57) = -2.83, *p* = .01). Infant cortisol levels were consistent with prior published studies in the same age range (e.g., Gunnar et al., 1996; Irwin et al., 2021; Jansen et al., 2010).

### Preliminary Analyses

**Prenatal internalizing symptoms.** In the EFA model, the two-factor solution failed to converge due to negative residual variance in anxiety symptoms. Given poor model fit from the one-factor solution in the EFA, χ^2^(9) = 92.94, *p* = .000, RMSEA = 0.24, 90% CI: 0.20, 0.29, CFI = 0.69, TLI = 0.48, SRMR = 0.09, prenatal anxiety and depressive symptoms could not be represented by a single higher-order factor. Instead, anxiety and depressive symptoms from each prenatal assessment were summed to calculate separate total scores of prenatal anxiety and depressive symptoms, respectively. Sum scores were used to capture greater variability in symptoms and provide a measure of total fetal exposure to maternal depressive and anxiety symptoms across pregnancy, rather than exposure at a single point of gestation (McNeish & Wolf, 2020). Moreover, sum scores are the most appropriate for calculating total scores in the context of missing data because there are less strict assumptions and do not change the substantive conclusions (Mazza et al., 2015; McNeish & Wolf, 2020).

**Social support.** In the EFA model, Chi-square difference tests indicated that the two-factor solution fit the data better than the one factor solution, χ^2^(8) = 92.96, *p* = .000, and the three-factor solution was not a better fit to the data than the two factor solution, χ^2^(7) = 8.31, *p* = .3058. Within the two-factor solution, one factor emerged consisting of five items reflecting available support from family as well as family closeness: ENRICHD Social Support (λ = 0.28), family instrumental support (λ = 0.63), frequency of family contact (λ = 0.62), family closeness (λ = 0.85), and family closeness not including spouse (λ = 0.86). However, the second factor, in which social support indicators loaded both negatively (ENRICHD Social Support: λ = -0.62; frequency of friend contact: λ = -0.69) and positively (friend instrumental support: λ = 0.63; friend closeness: λ = 0.81) on the factor, was not interpretable. The two factors were not significantly correlated, *p* > .05.

We conducted CFA to evaluate model fit for the first social support factor. Fit statistics indicated good fit to the data (χ^2^(6) = 2.48, *p* = .87; CFI = 1.00; TLI = 1.00; RMSEA = 0.000, 90% CI: [0.00, 0.05]; SRMR = 0.05). Residual indicator variances were not correlated. ENRICHD Social Support (λ* = 0.39), family instrumental support (λ* = 0.64), family contact (λ* = 0.63), family closeness (λ* = 0.83), and non-spousal family closeness (λ* = 0.88) loaded significantly onto the social support factor (*p*’s < 0.001). Therefore, one latent variable for social support, representing friend and family support, in pregnancy was used in primary analyses. Factor loadings for exploratory factor analysis and confirmatory factor analysis are visually presented in the Supplemental Materials.

### Primary Analyses

**Base Model.** First, we tested whether the association between maternal early life adversity and infant cortisol reactivity and recovery was mediated by maternal depressive and/or anxiety symptoms in pregnancy. Regression coefficients for the base model are presented in Table 3.

**Table 3:** Base Model Results

Infant Cortisol Responses at 1 month *Model fit*: χ^2^(8) = 12.29, *p* = .14; RMSEA = 0.06 [95% CI 0.00, 0.12], CFI = 0.98, TLI = 0.90, SRMR = 0.04				
**Outcome**	**Predictor**	**β**	**SE**	***p*** **-value**
Prenatal depressive symptoms	Maternal early life adversity	0.31	0.08	< 0.001***
Prenatal anxiety symptoms	Maternal early life adversity	0.14	0.08	0.071^
Infant cortisol reactivity	Maternal early life adversity	-0.04	0.11	0.743
	Prenatal depressive symptoms	-0.31	0.14	0.024*
	Prenatal anxiety symptoms	0.13	0.18	0.497
Infant cortisol recovery	Maternal early life adversity	-0.07	0.14	0.589
	Prenatal depressive symptoms	-0.04	0.17	0.802
	Prenatal anxiety symptoms	-0.02	0.21	0.939
**Indirect Effects**				
Maternal early life adversity → prenatal depressive symptoms → infant cortisol reactivity		-0.10	0.05	0.049*
Maternal early life adversity → prenatal anxiety symptoms → infant cortisol reactivity		0.02	0.03	0.525
Maternal early life adversity → prenatal depressive symptoms → infant cortisol recovery		-0.01	0.05	0.802
Maternal early life adversity → prenatal anxiety symptoms → infant cortisol recovery		-0.00	0.03	0.939
**Infant Cortisol Responses at 6 months** *Model fit*: χ^2^(8) = 6.72, *p* = .57; RMSEA = 0.00 [95% CI 0.00, 0.18], CFI = 1.00, TLI = 1.04, SRMR = 0.03				
**Outcome**	**Predictor**	**β**	**SE**	***p*** **-value**
Prenatal depressive symptoms	Maternal early life adversity	0.30	0.08	< 0.001***
Prenatal anxiety symptoms	Maternal early life adversity	0.13	0.08	0.105
Infant cortisol reactivity	Maternal early life adversity	-0.06	0.12	0.609
	Prenatal depressive symptoms	0.14	0.18	0.449
	Prenatal anxiety symptoms	0.41	0.16	0.010*
Infant cortisol recovery	Maternal early life adversity	0.13	0.12	0.267
	Prenatal depressive symptoms	-0.03	0.18	0.879
	Prenatal anxiety symptoms	0.38	0.16	0.018*
**Indirect Effects**				
Maternal early life adversity → prenatal depressive symptoms → infant cortisol reactivity		0.04	0.06	0.462
Maternal early life adversity → prenatal anxiety symptoms → infant cortisol reactivity		0.06	0.04	0.168
Maternal early life adversity → prenatal depressive symptoms → infant cortisol recovery		0.01	0.05	0.871
Maternal early life adversity → prenatal anxiety symptoms → infant cortisol recovery		-0.05	0.04	0.194

*Note.* Standardized model results are presented. Models adjusted for maternal depressive and anxiety symptoms at the time infant cortisol responses were measured, maternal ethnicity, per capita household income adjusted for cost of living at each study site, and maternal age.

^ *p* < .10; * *p* < .05; *** *p* < .001

***Infant cortisol responses at one month.*** The model examining infant cortisol responses to the heel stick at one month showed good fit (χ^2^(8) = 12.29, *p* = .14; RMSEA = 0.06 [95% CI 0.00, 0.12], CFI = 0.98, TLI = 0.90, SRMR = 0.04). Maternal early life adversity was unrelated to infant cortisol reactivity to or recovery from the heel stick at one month. However, maternal depressive symptoms in pregnancy significantly mediated predictions of infant cortisol reactivity to the heel stick at one month from maternal early life adversity. Maternal early life adversity positively predicted depressive symptoms in pregnancy and greater depressive symptoms were significantly associated with lower infant cortisol reactivity to the heel stick. Maternal early life adversity was not significantly associated with maternal anxiety symptoms in pregnancy and anxiety symptoms in pregnancy did not predict infant cortisol reactivity at one month. Neither maternal prenatal depressive nor anxiety symptoms predicted infant cortisol recovery at one month.

***Infant cortisol responses at six months.*** The model examining infant cortisol responses to the Still Face paradigm at six months also showed good fit (χ^2^(8) = 6.72, *p* = .57; RMSEA = 0.00 [95% CI 0.00, 0.18], CFI = 1.00, TLI = 1.04, SRMR = 0.03). Maternal early life adversity was not significantly associated with infant cortisol reactivity or recovery at six months. Maternal early life adversity positively predicted maternal prenatal depressive symptoms but not maternal prenatal anxiety symptoms. Maternal depressive symptoms in pregnancy did not significantly predict cortisol reactivity or recovery at six months. However, maternal anxiety symptoms in pregnancy positively predicted infant cortisol reactivity to the Still Face and inversely predicted cortisol recovery from the Still Face at six months, such that infants of women who reported greater anxiety symptoms in pregnancy showed greater reactivity and lower recovery.

**Moderation by social support.** We ran separate models testing (1) whether prenatal social support modified the association between maternal early life adversity and internalizing symptoms in pregnancy with infant cortisol responses at one month and six months and (2) whether prenatal social support modified the association between maternal internalizing symptoms in pregnancy and infant cortisol responses at one month and six months. Regression coefficients for each model are presented in the Supplemental Materials.

***Maternal early life adversity x social support in pregnancy.*** The models examining whether prenatal social support modified the association between maternal early life adversity and internalizing symptoms in pregnancy with infant cortisol responses to the heel stick at one month and to the Still Face at six months showed good fit (one month χ^2^(12) = 15.15, *p* = .23; RMSEA = 0.04 [95% CI 0.00, 0.09], CFI = 0.98, TLI = 0.93, SRMR = 0.04; six months χ^2^(24) = 20.55, *p* = .67; RMSEA = 0.00 [95% CI 0.00, 0.05], CFI = 1.00, TLI = 1.05, SRMR = 0.07). Prenatal social support did not significantly moderate the association between maternal early life adversity and depressive symptoms or anxiety symptoms in pregnancy in either model. Additionally, social support did not moderate the association between maternal early life adversity and infant cortisol reactivity or recovery at one month or six months.

***Prenatal internalizing symptoms x social support in pregnancy.*** The models examining whether prenatal social support modified the association between maternal internalizing symptoms and infant cortisol responses to the heel stick at one month and to the Still Face at six months showed good fit (one month χ^2^(14) = 19.20, *p* = .16; RMSEA = 0.05 [95% CI 0.00, 0.10], CFI = 0.98, TLI = 0.91, SRMR = 0.04; six months χ^2^(14) = 20.72, *p* = .11; RMSEA = 0.05 [95% CI 0.00, 0.10], CFI = 0.97, TLI = 0.86, SRMR = 0.04). Prenatal social support did not moderate the association of maternal prenatal depressive symptoms with infant cortisol reactivity or recovery at either study visit. Furthermore, social support did not modify the association between prenatal anxiety symptoms and infant cortisol reactivity or recovery at either study visit.

## Discussion

Childhood adversity is a replicated risk factor for psychopathology across the lifespan, including potentiating risk for future offspring. However, the pathways mediating these associations as well as potential resilience-promoting processes have not been reliably discerned. Guided by *developmental cascades* (Masten & Cicchetti, 2016) and prenatal programming models (Bowers & Yehuda, 2015; Roubinov et al., 2021; Davis & Narayan, 2020), the current study evaluated prenatal risk and resilience factors in the association between maternal early life adversity and infant stress regulation, a replicated biomarker of psychopathology risk (Gunnar, 1998; Koss & Gunnar, 2018). Specifically, we tested maternal depressive and anxiety symptoms during pregnancy as mediators of the association between maternal early life adversity and infant cortisol reactivity to and recovery from standardized stress paradigms at one month and six months of age. Furthermore, we tested social support in pregnancy as a moderator of the association between (1) maternal early life adversity and psychological distress in pregnancy and/or (2) maternal psychological distress in pregnancy and infant cortisol regulation.

Consistent with our hypothesis, maternal internalizing symptoms in pregnancy mediated the linkage of maternal early life adversity with dysregulated infant cortisol responses to stress. Specifically, there was a significant indirect effect of maternal early life adversity on cortisol reactivity to the heel stick at one month through maternal depressive symptoms. Although maternal childhood adversity predicts offspring biobehavioral stress reactivity (Hipwell et al., 2019; Bowers & Yehuda, 2015), a potent risk factor for poor outcomes across development (Koss & Gunnar, 2018), maternal early life adversity did not directly predict infant cortisol reactivity or recovery in the current study. Prior studies have also reported similar null associations (e.g., Bosquet Enlow et al., 2011; Brand et al., 2010). The indirect effect of maternal early life adversity on infant cortisol reactivity via maternal depressive symptoms in pregnancy is consistent with prior evidence indicating that early life adversity is associated with greater depressive symptoms in pregnancy (Racine et al., 2021) and that depressive symptoms in pregnancy predict infant cortisol responses to acute stressors (Howland et al., 2017; Bleker et al., 2020), including lower reactivity to acute stress (Galbally et al., 2019). Notably, the current study extends existing evidence insofar as uniquely testing maternal psychological distress in pregnancy as a mediator of the association between maternal early life adversity and infant stress regulation. Despite the potential adverse consequences associated with dysregulated cortisol regulation (e.g., blunted reactivity, elevated reactivity, slow recovery), including links with externalizing and internalizing symptoms (Koss & Gunnar, 2018), available interventions improve cortisol regulation in infants and young children (Slopen et al., 2014). Thus, cortisol regulation may represent an early emerging, modifiable intervention target to mitigate future risk for psychopathology.

Although maternal early life adversity predicted infant cortisol reactivity to the heel stick indirectly through depressive symptoms in pregnancy, this did not extend to infant cortisol reactivity at six months. Contrary to hypotheses, depressive symptoms were not associated with cortisol reactivity to the Still Face paradigm at six months. This pattern of results aligns with previous studies where prenatal stress predicted infant cortisol reactivity to stressors, but the strength and direction of the association depended on infant age and nature of the stressor (Tollenaar et al., 2011). In the first six months of life, attachment bonds and co-regulatory processes are rapidly developing all while the infant is particularly sensitive to postnatal environmental influences (Gee & Cohodes, 2021). Together, these factors may converge to alter physiological responses to stress, particularly during a relational stressor like the Still Face paradigm, thereby dampening the effect of prenatal depressive symptoms on infant cortisol reactivity. However, maternal anxiety symptoms in pregnancy predicted higher reactivity to and lower recovery from the Still Face, consistent with prior evidence linking maternal anxiety in pregnancy with infant stress regulation (Howland et al., 2017). Maternal early life adversity was positively, but non-significantly, associated with greater anxiety symptoms in pregnancy, which aligns with meta-analytic evidence reporting stronger effects of early life adversity on depressive than anxiety symptoms in pregnancy (Racine et al., 2021).

Anxiety and depressive symptoms often co-occur but may differentially affect fetal and offspring development. Importantly, depressive and anxiety symptoms were examined in the same analytical model in the current study, allowing for testing independent effects. We found that depressive symptoms were associated with lower cortisol reactivity whereas anxiety symptoms were associated with higher infant cortisol reactivity. This pattern is consistent with other studies where depressive and anxiety symptoms were independently associated with fetal behavioral reactivity (as measured by eye blink reactivity in Reissland et al., 2018). Although few studies have evaluated independent effects in the same study, some studies report similar patterns of results such that prenatal anxiety symptoms were associated with higher cortisol reactivity (Stroud et al., 2016) and lower recovery (Grant et al., 2009), whereas prenatal depressive symptoms were associated with blunted cortisol reactivity (Galbally et al., 2020) in infants.

Evidence on the biological mechanisms linking maternal prenatal distress to infant outcomes is limited; however, it has been hypothesized that different forms of distress operate through distinct physiological pathways to influence fetal development (Glover et al., 2010; Howland et al., 2017; Monk et al., 2019; O’Donnell & Meaney, 2017). This may be one reason for different independent effects of depressive and anxiety symptoms. Differences in independent effects may also be due to the nature of the stressor paradigms at each visit (physical stressor vs. social stressor; Gunnar et al. 2009; Jansen et al. 2010) and/or the dynamic developmental shifts in HPA axis regulation that occur in the first year of life (Gunnar et al., 1996; Jansen et al., 2010; Lewis & Ramsay, 1995). Ultimately, these findings suggest the need for further examination of independent effects of depressive and anxiety symptoms on infant development with particular focus on the potentially distinct biological pathways through which prenatal maternal depressive and anxiety symptoms may influence infant HPA axis regulation. It is also plausible that differences in the independent effects of depressive and anxiety symptoms are due to distinct links between each form of distress and parenting behaviors, formation of attachment bonds, and co-regulatory processes (e.g., Feldman et al., 2009; Hakanen et al., 2019). However, the current results were robust to statistical adjustment for maternal concurrent depressive and anxiety symptoms at the time of each cortisol assessment.

Prenatal mental health problems are a significant public health concern due to their prediction of physical and emotional well-being of both mothers and offspring across the lifespan (Center for Disease Control and Prevention, 2019b). Results from the current study add to growing evidence that early life adversity is a risk factor for depressive symptoms in pregnancy, perhaps operating through several plausible pathways. For example, women’s recollection of caregiving in their own childhoods become especially salient during pregnancy (Slade et al., 2009) which may confer heightened risk for mental health difficulties directly or indirectly by interacting with expected pregnancy-related stressors (Hammen et al., 2000; Narayan et al., 2017). Early experiences may also increase risk for psychological distress in pregnancy through biological embedding, such as changes to the nervous, endocrine, and immune systems (Danese & McEwen, 2012). Future work must consider potential biomarkers, such as HPA axis dysregulation (Seth et al., 2016), that may mediate the association of maternal early life adversity with internalizing symptoms to interrupt the cycle of risk.

The findings from the current study have important clinical implications. These results add to small but growing literature that the prenatal period is a time in which maternal early life experiences affect offspring development through physiological and psychosocial pathways (Roubinov et al., 2021). For example, maternal early life adversity was unrelated to infant cortisol reactivity, but indirectly predicted infant cortisol reactivity through maternal HPA axis functioning in pregnancy in prior studies (Thomas et al., 2018). Together with the current results, these findings suggest that maternal early childhood experiences may predict infant outcomes through maternal mental health and physiological functioning in pregnancy. Importantly, unlike early life experiences, maternal mental health in pregnancy is amenable to intervention. Moreover, the prenatal period may be an optimal period for intervention given frequent contact with the medical system and heightened developmental plasticity among expectant individuals and offspring (Davis & Narayan, 2020). Prenatal screening and intervention, particularly those including comprehensive life history strategies, may improve outcomes not only for mothers, but also for infants, and should be prioritized to help interrupt the intergenerational transmission of the consequences of early life adversity (Davis & Narayan, 2020). Nonetheless, the current study only examined one plausible pathway linking maternal early life adversity to infant outcomes; postnatal environmental factors, including parenting behaviors and bidirectional co-regulatory processes (Sameroff, 2010), may also relate to maternal early life adversity, internalizing symptoms, and infant stress regulation. Such pathways warrant investigation in future studies.

Contrary to hypotheses, social support did not significantly moderate predictions of infant cortisol regulation from maternal early life adversity or maternal internalizing symptoms. This aligns with previous evidence where social support did not modify associations between early life adversity and internalizing symptoms in pregnancy (e.g., Racine et al., 2020; Wajid et al., 2020). There may be several reasons for these null results. Moderation by social support in prior studies may reflect social buffering of the maternal HPA axis (Giesbrecht et al., 2013; Thomas et al., 2017, 2018), a process in which the availability of support reduces the activity of stress-responsive neurobiological systems (Gunnar & Hostinar, 2015). For example, one study found that social support moderated the association between maternal early life adversity and HPA axis function in pregnancy, and HPA axis function in turn predicted infant cortisol reactivity. Therefore, the degree to which social support influences infant cortisol reactivity may depend on the extent to which social support buffers maternal neurobiological responses to stress in pregnancy (Howland et al., 2017). It is also important to note that prior studies operationalized social support as “good, ongoing interpersonal support” from their partner (e.g., Thomas et al., 2017, 2018) which differs from the present study. Future research with repeated measures of maternal prenatal cortisol, internalizing problems, and social support are needed to rigorously test whether social support buffers against the adverse effects of early life adversity via HPA axis regulation in pregnancy. Additionally, the current study only examined social support from friends and family and did not differentiate between types of social support. Valid and reliable assessment of different types of social support may be especially critical for women who experienced early life adversity (Racine et al., 2020). Finally, given that tests of interactions, especially in non-experimental designs, are vulnerable to Type I and Type II error, the modest sample size may have been underpowered to detect a significant interaction.

Despite these null findings, examining psychosocial moderators of this intergenerational process in the prenatal period represents a novel direction for developmental psychopathology research. Limited work has assessed protective effects of psychosocial factors during pregnancy for women with histories of early life adversity. While the few prior studies that examined prenatal protective factors for individuals with a history of early life adversity have focused on social support (see Atzl et al., 2019), it may be that there are other salient protective factors that promote resilience for mothers and infants. For example, personal resources such as mastery, self-esteem, and spirituality protected against depressive symptoms in the perinatal period (Julian et al., 2021) and lifestyle behaviors may play an important role in the intergenerational transmission of early life adversity (De Weerth, 2018).

### Strengths and Limitations

The longitudinal design of this study enabled a rigorous methodological test of developmental cascades and mediating effects (Masten & Cicchetti, 2016). The study was also well-positioned to isolate the unique effects of maternal preconception stress (i.e., early life adversity and internalizing symptoms) on infant cortisol reactivity from a prenatal programming perspective with its conservative control of postpartum psychological distress. The infant HPA axis develops rapidly across the first six months of life; including repeated measures of cortisol reactivity using a physical stressor at one month and a social stressor at six months allowed for assessment of maternal early life and prenatal influences on HPA axis responses in the first six months of life, a critical period for the calibration of the stress response system. Future studies should test developmental continuity and change in larger samples using growth modeling approaches, for example, to rigorously test how prenatal stress relates to continuity and change in infant cortisol regulation across the first six months of life.

This study also had important limitations. First, retrospective reports of maternal early life experiences are vulnerable to reporting biases (Baldwin et al., 2019). Nevertheless, this method overall is valid, correlates significantly with prospective accounts of objective evidence, and recall bias does not significantly affect estimates of the impact of child adversity on mental health (Brewin, Andrews, & Gotlib, 1993; Fergusson, Horwood, & Boden, 2011). Additionally, the majority of mothers identified as White and were relatively well-educated and partnered, which may not generalize to key constituencies based on socioeconomic status, race-ethnicity, and cultural identification. Moreover, it was beyond the framework of the current study to evaluate the mediating role of recent trauma, stressful life events, or perceived stress during pregnancy, which may contribute to the prenatal programming of infant physiology (Sosnowski et al., 2018). Given the sample size and model complexity, we were underpowered to test for sex differences in the pathways linking early life adversity to infant stress regulation. Sensitivity of the developing fetus to risk and salutary processes may differ by sex (e.g., Sandman et al., 2013). Another plausible explanation for the findings could be passive gene-environment correlation (i.e., shared genes between the mother and infant); however, results are consistent with prior literature that use cross-fostering in animal studies (e.g., Abe et al., 2007). Finally, it was outside of the scope of this study to evaluate how salient postnatal environmental factors, namely parent-infant co-regulatory processes (Sameroff, 2010), contribute to and/or modify links between maternal early experiences, internalizing symptoms in pregnancy, and infant cortisol regulation.

## Conclusion

The current study was guided by theoretical frameworks of prenatal programming and developmental cascades of risk and resilience. The findings suggested that maternal depressive symptoms during pregnancy are a key pathway linking maternal early life adversity to infant cortisol reactivity at one month. However, prenatal social support did not modify associations of maternal early life adversity with internalizing symptoms in pregnancy or associations of internalizing symptoms in pregnancy with infant stress regulation. Given that infant HPA axis dysregulation is an early emerging marker of risk for psychopathology, psychosocial factors in the prenatal environment may represent modifiable targets of prevention and intervention. Improved screening of maternal history of adversity and psychological distress along with increased access to mental health services during pregnancy might help to mitigate the downstream consequences of maternal early life adversity.

### Electronic Supplementary Material

Below is the link to the electronic supplementary material.

Supplementary Material 1

## Data Availability

The data are not available to the public because they contain confidential mental health information of participants.

## Footnotes

1 Participants in the current analytic sample (n = 162) completed significantly more education, reported a significantly higher per capita income, were significantly older, and were less likely to identify as Hispanic/Latina compared to the full sample (n = 233).

2 When evaluating the one-month and six-month outcomes in the same model (consistent with the developmental cascades framework), the results remained the same as in the individual models but model fit was not as strong. Therefore, we present the models with each time point examined separately.

## Code Availability

Available upon request.
