# Supplementary material for: Maternal Early Life Adversity and Infant Stress Regulation: Intergenerational Associations and Mediation by Maternal Prenatal Mental Health
Source: Res Child Adolesc Psychopathol. Author manuscript; Available in PMC 2024 Jun 1. (PMC10258218; doi:10.1007/s10802-022-01006-z)
Supplement: Supplementary Material [file NIHMS1879845-supplement-Supplementary_Material.docx]

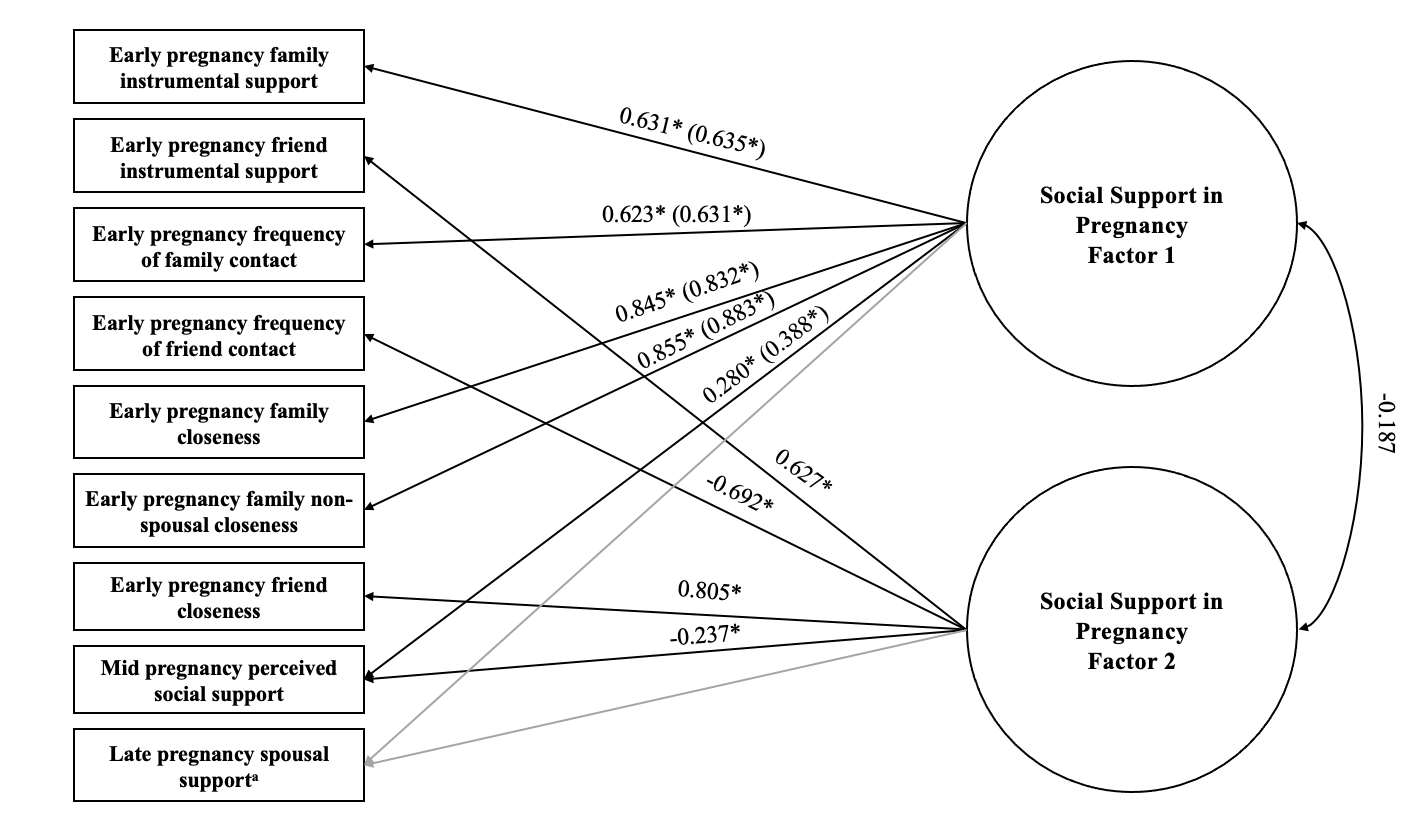


*Note.* The two-factor solution was identified as the best fit with exploratory factor analysis (χ^2^(19) = 26.616, *p* = 0.1139; CFI = 0.977; TLI = 0.956; RMSEA = 0.050, 90% CI: [0.000, 0.091]; SRMR = 0.037). Factor 2 was uninterpretable (i.e., had both negative and positive factor loadings) therefore was not used in primary analyses. Factor 1 was subsequently confirmed with confirmatory factor and used in primary analyses. Factor loadings from exploratory factor analysis for all variables that significantly loaded onto a factor are presented and factor loadings from confirmatory factor analysis of the first factor are shown in parentheses.

^a^ Late pregnancy spousal support did not significantly load onto Factor 1 (0.023) or Factor 2 (-0.046).

| **Supplemental Table 2. Mediated Moderation Results** | | | | |
| --- | --- | --- | --- | --- |
| **Infant Cortisol Responses at 1 month**  *Model fit:* χ^2^(12) = 15.15, *p* = .23; RMSEA = 0.04 [95% CI 0.00, 0.09], CFI = 0.98, TLI = 0.93, SRMR = 0.04 | | | | |
| **Outcome** | **Predictor** | **β** | **SE** | ***p*-value** |
| Prenatal depressive symptoms | Early life adversity x social support | 0.093 | 0.095 | .327 |
| Prenatal anxiety symptoms | Early life adversity x social support | 0.062 | 0.114 | .587 |
| Infant cortisol reactivity | Maternal early life adversity | 0.038 | 0.168 | .822 |
|  | Prenatal depressive symptoms | -0.254 | 0.162 | .116 |
|  | Prenatal anxiety symptoms | 0.106 | 0.201 | .599 |
|  | Social support | 0.129 | 0.174 | .458 |
|  | Maternal early life adversity x social support | 0.031 | 0.186 | .867 |
| Infant cortisol recovery | Maternal early life adversity | 0.039 | 0.174 | .822 |
|  | Prenatal depressive symptoms | -0.073 | 0.179 | .684 |
|  | Prenatal anxiety symptoms | -0.012 | 0.263 | .965 |
|  | Social support | -0.107 | 0.199 | .592 |
|  | Early life adversity x social support | 0.040 | 0.197 | .840 |
|  | Maternal early life adversity x social support | 0.040 | 0.197 | .840 |
| **Infant Cortisol Responses at 6 months**  *Model fit:* χ^2^(12) = 20.55, *p* = .67; RMSEA = 0.00 [95% CI 0.00, 0.05], CFI = 1.00, TLI = 1.05, SRMR = 0.07 | | | | |
| **Outcome** | **Predictor** | **β** | **SE** | ***p*-value** |
| Prenatal depressive symptoms | Early life adversity | 0.267 | 0.102 | .009** |
|  | Social support | -0.171 | 0.100 | .087^ |
|  | Early life adversity x social support | 0.078 | 0.095 | .412 |
| Prenatal anxiety symptoms | Early life adversity | 0.058 | 0.092 | .526 |
|  | Social support | -0.219 | 0.129 | .091^ |
|  | Early life adversity x social support | 0.047 | 0.112 | .674 |
| Infant cortisol reactivity | Maternal early life adversity | -0.014 | 0.152 | .924 |
|  | Prenatal depressive symptoms | -0.028 | 0.194 | .886 |
|  | Prenatal anxiety symptoms | 0.391 | 0.211 | .064^ |
|  | Social support | -0.039 | 0.191 | .836 |
|  | Early life adversity x social support | 0.123 | 0.151 | .418 |
| Infant cortisol recovery | Maternal early life adversity | -0.287 | 0.181 | .112 |
|  | Prenatal depressive symptoms | 0.001 | 0.187 | .997 |
|  | Prenatal anxiety symptoms | -0.346 | 0.146 | .017* |
|  | Social support | -0.054 | 0.171 | .750 |
|  | Early life adversity x social support | -0.279 | 0.157 | 0.075 |
| *Note.* Standardized model results are presented. Models adjusted for maternal depressive and anxiety symptoms at the time infant cortisol responses were measured, maternal ethnicity, per capita household income adjusted for cost of living at each study site, and maternal age.  ^ p < .10; * p < .05; ** p < .01 | | | | |

| **Supplemental Table 3. Moderated Mediation Results** | | | | |
| --- | --- | --- | --- | --- |
| **Infant Cortisol Responses at 1 month**  *Model fit:* χ^2^(14) = 19.20, *p* = .16; RMSEA = 0.05 [95% CI 0.00, 0.10], CFI = 0.98, TLI = 0.91, SRMR = 0.04 | | | | |
| **Outcome** | **Predictor** | **β** | **SE** | ***p*-value** |
| Prenatal depressive symptoms | Maternal early life adversity | 0.247 | 0.091 | .006** |
| Prenatal anxiety symptoms | Maternal early life adversity | 0.086 | 0.075 | .253 |
| Infant cortisol reactivity | Maternal early life adversity | 0.085 | 0.166 | .606 |
|  | Prenatal depressive symptoms | -0.275 | 0.169 | .103 |
|  | Prenatal anxiety symptoms | 0.120 | 0.201 | .551 |
|  | Social support | 0.177 | 0.185 | .606 |
|  | Depressive symptoms x social support | 0.044 | 0.195 | .822 |
|  | Anxiety symptoms x social support | -0.035 | 0.208 | .865 |
| Infant cortisol recovery | Maternal early life adversity | 0.005 | 0.154 | .974 |
|  | Prenatal depressive symptoms | -0.070 | 0.186 | .705 |
|  | Prenatal anxiety symptoms | 0.004 | 0.259 | .987 |
|  | Social support | -0.086 | 0.217 | .690 |
|  | Depressive symptoms x social support | -0.283 | 0.290 | .330 |
|  | Anxiety symptoms x social support | 0.055 | 0.327 | .867 |
| **Infant Cortisol Responses at 6 months**  *Model fit:* χ^2^(14) = 20.72, *p* = .11; RMSEA = 0.05 [95% CI 0.00, 0.10], CFI = 0.97, TLI = 0.86, SRMR = 0.04 | | | | |
| **Outcome** | **Predictor** | **β** | **SE** | ***p*-value** |
| Prenatal depressive symptoms | Maternal early life adversity | 0.240 | 0.091 | .008** |
| Prenatal anxiety symptoms | Maternal early life adversity | 0.078 | 0.075 | .303 |
| Infant cortisol reactivity | Maternal early life adversity | -0.073 | 0.129 | .572 |
|  | Prenatal depressive symptoms | 0.137 | 0.207 | .508 |
|  | Prenatal anxiety symptoms | 0.420 | 0.214 | .054^ |
|  | Social support | -0.036 | 0.168 | .833 |
|  | Early life adversity x depressive symptoms | 0.114 | 0.261 | .663 |
|  | Early life adversity x anxiety symptoms | 0.108 | 0.343 | .752 |
| Infant cortisol recovery | Maternal early life adversity | -0.158 | 0.156 | .310 |
|  | Prenatal depressive symptoms | 0.045 | 0.197 | .819 |
|  | Prenatal anxiety symptoms | -0.416 | 0.165 | .012* |
|  | Social support | -0.078 | 0.184 | .672 |
|  | Early life adversity x depressive symptoms | 0.085 | 0.253 | .737 |
|  | Early life adversity x anxiety symptoms | -0.256 | 0.307 | .405 |
| *Note.* Standardized model results are presented. Models adjusted for maternal depressive and anxiety symptoms at the time infant cortisol responses were measured, maternal ethnicity, per capita household income adjusted for cost of living at each study site, and maternal age.  ^ p < .10; * p < .05; ** p < .01 | | | | |
